# Skills and abilities to thrive in remote work: What have we learned

**DOI:** 10.3389/fpsyg.2022.893895

**Published:** 2022-12-19

**Authors:** Jonn B. Henke, Samantha K. Jones, Thomas A. O’Neill

**Affiliations:** Department of Psychology, University of Calgary, Calgary, AB, Canada

**Keywords:** remote work, individual differences, qualitative, thematic analysis, telework, work from home, skills

## Abstract

The COVID-19 pandemic led to a rapid acceleration in the number of individuals engaging in remote work. This presented an opportunity to study individuals that were not voluntarily working remotely pre-pandemic and examine how they adapted and learned to achieve success in a remote work environment, at an organization that did not have substantial prior experience managing remote work. We used a semi-structured interview process to interview participants (*n* = 59) who occupied both Individual Contributor and Leadership levels at an organization and broadly representative across several important demographic characteristics. We asked participants to discuss what factors at individual, team, and organizational levels contributed positively toward their remote work experience, which factors presented challenges to remote work, and what could be done to ensure success with remote work in the future. Interviews were analyzed utilizing a thematic analysis approach and summarized into common themes pertaining to factors that influence success in a remote working environment. Themes were used to identify specific skillsets particularly relevant to remote work that would benefit from training, as well as important organizational culture changes and policies needed to support remote workers and ensure their success. We present these and other findings in relation to current research and provide recommendations for practitioners.

## Introduction

In March 2020, the coronavirus (COVID-19) pandemic resulted in a rapid shift in the way employees all over the world engaged in employment as governments encouraged, or mandated, work from home orders to slow the spread of the virus. As a result, employees who were working in a traditional face-to-face setting, and perhaps had never worked remotely before, experienced an unexpected mass shift to working from home. A key challenge raised by remote work is that of technology-mediated communication, which requires employees to adapt conventional face-to-face, in-person, and in-office work interactions to the virtual realm (McLarnon et al., 2019). Technology-mediated communication requires that individuals, teams, and leaders utilize the right medium, at the right time, for the right message (i.e., the temporal matching of media richness to the message; Gilson et al., 2021). While tremendous laboratory and field-based research exists on virtual communication in general (e.g., Dennis et al., 2008), no previous adoption of remote work occurred so quickly, with so little preparation, at the scale created by the COVID-19 pandemic.

Remote work has been extensively studied in the past, alongside similar concepts or terminologies such as telework, telecommuting, or virtual teaming, which are all seeing growing interest given the current landscape (e.g., Gilson et al., 2021; Roulin et al., 2021). Each of these terms, among various others, have seen inconsistent usage; some have attempted to differentiate between them (e.g., Allen et al., 2015), while others make linkages across the core concepts to coordinate research efforts across fields (Raghuram et al., 2019). As such, keeping in mind consideration of the past literature and the nature of remote work employed during the early days of the COVID-19 pandemic, we adopt the European Foundation for the Improvement of Living and Working Conditions’ (Eurofound, 2021) telework definition: “… a form of organizing and/or performing work, using information technology, in the context of an employment contract/relationship, where work, which could also be performed at the employers premises, is carried out away from those premises on a regular basis.”

For many organizations, switching to such an unfamiliar model of business operation was unprecedented and unplanned for, with some surveys indicating that only 12% of organizations were prepared for the changes brought by COVID-19 (Gartner, 2020). Despite this, many workers and organizations found success or even thrived in a remote work environment. Research has shown that remote workers can balance at-home demands (e.g., cleaning, childcare) without significant impact upon work performance (Anderson and Kelliher, 2020; Rofcanin and Anand, 2020), and Baudot and Kelly (2020) found many workers perceived themselves to be more productive working from home than in their prior arrangements, and desired to continue working remotely indefinitely. Indeed, analysis performed by McKinsey and Company (Lund et al., 2020) concluded that more than 20% of the workforce could spend 3–5 days of the week working remotely without any significant productivity loss to organizations.

These trends clearly indicate that there is both the potential and the demand for remote work to continue at a larger scale than ever seen before, and most anticipate remote work to persist after the ‘Return to Office,’ whether in the form of hybrid arrangements (Mitchell and Brewer, 2021), or as fully remote. In the academic literature, research indicates that the experiences of remote workers are affected by a wide range of personal and organizational factors, such as needing to adjust to the higher degree of autonomy and independence that remote work generally affords, or the challenges with needing to communicate only with the technology available to facilitate task progress and coordination with co-workers (Van Zoonen et al., 2021, Babapour Chafi et al., 2022). Many different theories and perspectives have been used to frame this body of research. For example, Trait Activation Theory proposes that workers will respond uniquely to the environment they are in based off of their unique personalities, skills, and experience (Tett and Guterman, 2000). In a remote work environment, personality traits such as Conscientiousness and Honesty were found to relate to the extent of cyberslacking – using the internet for non-work purposes during company time – that remote workers engage in, as they work in an environment which can make it easier to engage in such unproductive behavior unnoticed (O’Neill et al., 2014). The virtual environment can also influence the skills and behaviors remote workers need to leverage to be successful, linking to person-organization fit theory (Kristof, 1996). For example, the virtual environment requires additional emphasis on specific skills, such as communicating with technology (Varty et al., 2017), thus requiring different approaches from managers and leadership (Charlier et al., 2016; Maduka et al., 2018). Poor organizational support and communication such as inadequate resources (e.g., technology and software) and unclear direction from top management may foster an environment which undermines remote workers commitment and exacerbate social isolation in a remote work environment (Van Zoonen and Sivunen, 2021). Other remote workers may be juggling their work duties alongside childcare at home, which can influence aspects of engagement and job performance, and the timeliness of employees’ communications (Toscano and Zappalà, 2021).

Despite these various challenges researchers have investigated, especially in the wake of pandemic restrictions, research has shown that many individuals may prefer remote work, perform better while working remotely, and report greater work-life wellness (Aczel et al., 2021). However, remote workers and the leaders and managers of remote workers face various communication challenges that need to be better understood in order to form guidelines and recommendations that can better address those barriers to success. For example, the environment remote workers operate in may influence negative emotions to be suppressed instead of being expressed (Glikson and Erez, 2013), and may rely more on forms of communication where the intent and emotionality of their communication are at greater risk of being misinterpreted (Cheshin et al., 2011). These can cause problems such as misinterpreting the root cause of a remote worker’s inadequate performance (e.g., assuming a remote worker is slacking on certain tasks, when in actuality their workload has been too high and they are mentally overtaxed), or vice versa, remote workers not having an accurate understanding of what their organization expects of them from a performance standpoint to begin with. Barriers to success could fall through the cracks and fail to be discussed and addressed in such an environment. In light of the unique environment imposed by remote work compared to a traditional workplace and the large population of new remote workers with limited prior experience, it is vital to consider what we have learned with respect to remote work to success during the pandemic, and what can be transferred to a post-pandemic environment. By learning from the experiences of these new remote workers, and how they adapted to remote working, we can provide recommendations to organizations of how they can better communicate and coordinate with remote workers to address the challenges they face, and remove barriers to individual success and organizational wide goals.

For our current research, we were presented with the opportunity to interview corporate employees in a 100,000-employee healthcare organization, across geographies, functions, and levels of the organization. 20,000 of these individuals in corporate roles became remote workers instantly as the pandemic forced imminent lockdowns. Our interest was in identifying aspects of organizational culture, team interactions, and the skills and behaviors of workers as they adapted to remote work. By exploring a diverse range of these experiences, we sought to gain further understanding on what unique problems and needs remote workers have, and how to better support them in the future – from their individual roles and needs, to team interactions, and up to the context of the wider organization and its culture as a whole. We believe that there could be many lessons to be learned from the rapid shift to remote work and the individual differences, behaviors and routines, and organizational supports that helped employees remain successful in their role should be delineated. Accordingly, we report on a qualitative study in which we examined the following research question:

*Research Question:* What lessons can we learn from remote work experiences during the pandemic – that relied almost completely on technology-mediated communication in order to perform collective work tasks – that can be used to provide input into how remote work may be successful in the future?

## Materials and methods

### Participants

Following the recommendations put forth by Yin (2015), we conducted semi-structured interviews with 59 staff from a large Canadian healthcare organization between June and July 2021 (Table 1). Participants were recruited using a snowballing approach where we contacted members of a diverse and organizationally representative working group and asked them to nominate members of their department who might be interested in participating. The researchers then contacted the potential participants and invited them to schedule a time for an interview if they were interested in sharing their remote work experiences. Of the 71 potential participants we contacted, 59 participated representing an approximately 83% response rate. Our sample captured employees working across numerous organizational functions including: human resources including staffing, employee benefits and retirement, employee education and learning, executives, legal services, finances, technology support, and non-patient facing healthcare services (e.g., managers, administrative assistants in clinical settings). Current position tenure ranged from 1 month to over 20 years capturing the remote work perspectives of experienced employees as well as employees that were on-boarded while working remotely. Many participants (*n* = 26) noted that the geographically dispersed nature of the organization meant they often interacted with their colleagues virtually even when they were previously in the office which aided the transition to remote work. However, all were located in the same time zone.

**TABLE 1:** Frequencies for demographic variables.

Demographic		Frequency (%)
Position Type		
	Leader	35 (59)
	Individual Contributor	24 (41)
Remote Work Experience		
	Only since COVID-19	20 (34)
	Sporadically prior to COVID-19 (e.g., when sick/traveling)	24 (41)
	Consistently prior to COVID-19 (e.g., part-time)	15 (25)
Age		
	30 years and under	1 (2)
	31–40 years	9 (15)
	41–50 years	23 (39)
	Over 50 years	24 (41)
	Did not disclose	2 (3)
Gender		
	Female	39 (66)
	Male	19 (32)
	Did not disclose	1 (2)
Marital status		
	Married	46 (78)
	Common Law/Long-term partner	2 (3)
	Divorced	3 (5)
	Single	4 (7)
	Did not disclose	4 (7)
Dependents		
	One or more	21 (36)
	None	38 (64)

N = 59. Percentages in brackets represent the percentage of the total sample.

### Procedure

The interviews aimed to balance participants’ reflections on rapid learning experiences when transitioning to remote work during the COVID-19 pandemic, while also identifying key skills, behaviors, cultural shifts, and organizational supports that would be required to promote successful remote (full-time out of office) and hybrid remote (part-time out of office) work arrangements moving forward. Our interview guide broadly focused on questions related to the participants’ employment background (e.g., how long have you been utilizing remote work and to what degree?), their positive and negative reflections on remote work experiences (e.g., what behaviors, actions, or routines did you utilize that helped you be successful at remote working?), and how these experiences can inform remote work moving forward (e.g., what skills do you think need to be developed in order to effectively work remotely?).

### Analytic approach

Participant interviews were transcribed using the closed captioning function of Zoom during the interview, and no audio recordings were saved to protect the confidentiality of the participants. Interview transcripts were then reviewed by the interviewer, correcting errors, and removing identifying information. Two independent coders analyzed the data following Braun and Clarke’s (Braun and Clarke, 2012; Clarke and Braun, 2013) six-step process for thematic analysis in NVivo. Using a subset of 10 articles, the coders read and re-read the transcripts to become familiar with the data, followed by open coding to identify key concepts in the data. The coders then generated themes from the codes, reviewed, and refined the themes, and produced comprehensive definitions for each theme. This process was repeated with an additional 10 articles to finalize the coding framework and obtain a Cohen’s kappa coding agreement of 0.84, with percent agreement ranging from 98 to 100%. The first coder then analyzed the remaining transcripts with the second coder checking a random subset of interview transcripts for agreement. All discrepancies were solved through discussion.

## Results

**FIGURE 1:**
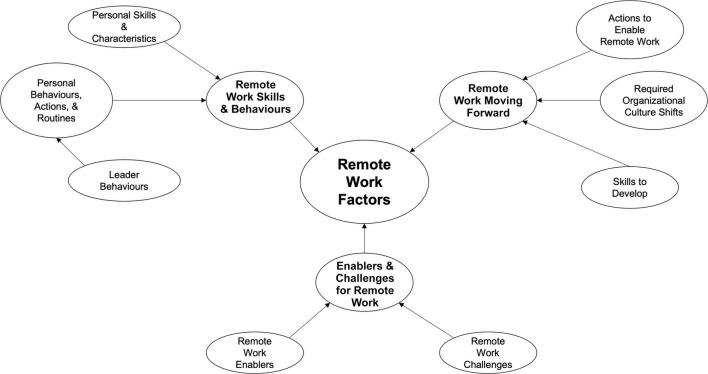
Thematic map of meta-themes and themes.

Our coding of the interview transcripts produced 59 codes that cover three major meta-themes – remote work skills and behaviors, enablers and challenges for working remotely, and remote work moving forward – each broken down into further themes (Table 2). The relationships between the meta-themes and themes can be found in the thematic map in Figure 1. Below, we highlight some of the primary considerations for each theme as well as exemplary quotes from the unique leader and individual contributor perspective.

**TABLE 2:** Summary of interview codes and themes.

Theme/Meta-theme	Code	Leader (*N* = 35) *n* (%)	IC (*N* = 24) *n* (%)	Total references

Remote Work Skills and Behaviors				
Personal Skills and Characteristics				
	Technology literacy	17 (49)	6 (25)	30
	Being independent	8 (23)	6 (25)	19
	Communication	9 (26)	5 (21)	20
	Strong work ethic	10 (29)	7 (29)	24
	Ability to manage distractions	4 (11)	8 (33)	14
	Time management	8 (23)	4 (17)	22
	Personality (Extraversion)	8 (23)	3 (13)	16
	Proactivity	0 (0)	3 (13)	3
	Supporting coworkers	4 (11)	3 (13)	10
Personal Behaviors, Actions, and Routines				
	Setting a normal work schedule	20 (59)	22 (92)	88
	Being intentional about interactions	14 (40)	6 (25)	39
	Creating a dedicated workspace	13 (37)	5 (21)	24
	Time management	12 (34)	7 (29)	26
	Checking in with colleagues	13 (37)	5 (21)	34
	Engaging in informal team activities	12 (34)	6 (25)	21
	Adapted resources used	10 (29)	2 (8)	16
Leader Behaviors				
	Enabling			
	Attentive to employee well-being	3 (9)	9 (38)	12
	Being available	3 (9)	6 (25)	10
	Check-ins	2 (6)	11 (46)	17
	Flexibility and providing supports	8 (23)	16 (67)	42
	Good communication	3 (9)	5 (21)	9
	Challenging			
	Poor communication, direction	3 (9)	7 (29)	25

**Enablers and Challenges for Working Remotely**				

Remote Work Enablers				
	Organizational Culture			
	Supporting Staff	18 (51)	14 (58)	43
	Positive perception of remote work	14 (40)	10 (29)	33
	Value-focused organization	8 (23)	4 (17)	16
	Embracing geographically dispersed teams	4 (11)	2 (8)	11
	Healthier life	8 (23)	10 (42)	32
Remote Work Challenges				
	Relationship building and maintenance	28 (80)	13 (38)	70
	Physical technology and equipment	20 (57)	9 (26)	53
	On-boarding and succession planning	21 (60)	10 (29)	52
	Connectivity and Wi-Fi	17 (49)	10 (29)	39
	Concerns of fairness and stigma	15 (43)	9 (26)	39
	Having a dedicated workspace	15 (43)	8 (33)	35
	Communication	15 (43)	8 (33)	39
	Performance management	16 (46)	6 (25)	32
	Work-life balance	13 (54)	8 (33)	32
	Work scheduling and time management	9 (26)	5 (21)	23
	Staying motivated	5 (14)	6 (25)	16

	Management visibility	6 (17)	3 (13)	11

**Remote Work Moving Forward**				

Skills to Develop				
	Managing virtually	19 (54)	8 (33)	41
	Technology literacy	18 (51)	7 (29)	32
	Time management	10 (29)	14 (58)	35
	Maintaining rapport virtually	15 (43)	6 (25)	34
	Communication	11 (31)	8 (33)	25
	Virtual soft skills	11 (31)	3 (13)	24
	Self-management	7 (20)	6 (25)	17
Required Organizational Culture Shifts				
	Trust in remote workers	19 (54)	11 (46)	58
	‘Camera-on’ culture	10 (29)	3 (13)	17
	Respect for work-life balance	3 (9)	1 (4)	6
	Maintaining changes	17 (49)	12 (50)	49
Actions to enable remote work				
	Provide technology and equipment	26 (74)	15 (63)	74
	Clear policies and procedures	25 (71)	15 (63)	76
	Listen to employee wants/needs	21 (60)	18 (75)	68
	Maintain office access	19 (54)	11 (46)	46
	Remote work training	17 (49)	7 (29)	30
	Communicating role expectations	17 (49)	6 (25)	46
	Clear cost expectations	12 (34)	4 (17)	21
	Relationship building opportunities	7 (20)	7 (29)	24
	Improve IT support	9 (26)	6 (25)	30

Total N = 59. IC, Individual Contributor. Percentages in brackets represent the percentage of the subsample (leaders or ICs) that were classified as contributing one or more statements to the code. Total References indicates the total number of statements coded under the code.

### Remote work skills and behaviors

#### Skills

Largely, participants, albeit leaders (*n* = 17; 49%) more than Individual contributor(s) (ICs) (*n* = 8; 33%), said they used many of the same skills working remotely as they did in the office (*n* = 25; 42%), adopting a ‘same skills, different environment’ mindset. Many participants attributed this to their longstanding reliance on technology to communicate with an increasingly geographically dispersed team: “*nothing’s changed really about my work, except for where I’m sitting. So, the work, the work has always been through Skype*” (BM2311, leader).

Despite many similarities in the types of tasks participants were engaging in, the majority of participants were able to identify at least one specific skill or characteristic that aided them in successfully working remotely (Table 2). Leaders most frequently cited skills such as technology literacy (*n* = 17; 49%), communication (*n* = 9; 26%), and work ethic (*n* = 10; 29%), perhaps reflecting their need to remain connected with their team and engage in role modeling behaviors: *“you have to be able to focus. You cannot become distracted. You cannot multitask. You need to be able to understand that, although you’re not face to face in the boardroom, you’re in a meeting”* (HD0801, leader). The importance of maintaining focus when working remotely was also supported by ICs as the ability to manage distractions was the most endorsed skill supporting remote work success (*n* = 8; 33%). For some, their home environment removed many office-specific distractions and allowed them to improve productivity while others, particularly in the context of the pandemic, had to leverage their distraction management skills to manage competing demands during the workday. Regardless, “*you have to be able to focus yourself because there’s still quite a bit of stimulus to focus you with our technology, text messages, instant messages. Like you’re still stimulated. It’s just different than a traditional workplace”* (JD1702, IC).

#### Behaviors

Overwhelmingly, participants cited setting a normal work schedule as being the most beneficial behavior for successful remote work (*n* = 42; 71%), especially for ICs (*n* = 22; 92%). This included waking up at a consistent time, getting ready normally (e.g., showering, putting on work clothes, having breakfast), setting break times, and ending their workday at their usual time. For many, settling into a regular remote workday took some time but ultimately, supported their long-term well-being: “*And now as we’re, you know, working through it [the pandemic] further we recognize that we don’t have to always be there and setting some boundaries is important for work life balance as well*” (BF0511, leader). Similarly, creating a dedicated workspace (leaders: *n* = 13, 37%, IC: *n* = 5, 21%) and engaging in time management behaviors (leaders: *n* = 12, 34%, IC: *n* = 7, 29%) were also identified as key factors in successful remote working.

Behaviors that supported social and professional networks and helped leaders and ICs stay connected were also important for supporting remote work practices. Participants commented how they had to be more intentional about their interactions with others to maintain their relationships in lieu of traditional ‘water cooler’ chats (leaders: *n* = 14, 40%, IC: *n* = 6, 25%). Checking in with colleagues (leaders: *n* = 13, 37%, IC: *n* = 5, 21%) and engaging in informal team activities (leaders: *n* = 12, 34%, IC: *n* = 6, 25%) were also vital to maintaining a productive and cohesive work team. For example, one team “*recognized that we have a stronger need, not at team meetings where we’re talking about business of the group, but separate meetings to just have those social connections, where we can talk about what’s going on in our lives, over and above our work lives”* (LA 1601, leader) citing that these types of connections spillover into an understanding of colleagues’ capabilities and needs.

In addition to general remote work behaviors, we explored enabling and challenging behaviors that were specific to being in a leadership position. Overall, participants largely had positive experiences with their leaders and leaders they worked alongside when transitioning to remote work, referencing behaviors such as providing flexibility and supports (IC: *n* = 16, 67%), regular but appropriate check-ins (IC: *n* = 11, 46%), and being attentive to employee well-being (IC: *n* = 9, 38%). Participants also mentioned the availability of leaders (IC: *n* = 6, 25%) and leaders’ increased comfort with remote work as enabling factors (IC: *n* = 2, 8%). Despite 15 (63%) ICs not having any negative behaviors to report, seven (29%) participants found that their leaders engaged in poor communication behaviors (e.g., too many meetings or calls, micromanaging) or failed to provide clear direction. One participant reflected on how ineffective communication could stem from a poor recognition of appropriate boundaries from leaders: “*she’s [my leader] not angry when you’re not available but she’s just notorious for like just calling and calling and calling everybody. It doesn’t matter if your light is green and available, or it says away or whatever, she’s calls*” (AH1809, IC).

### Current enabling and challenging factors for working remotely

#### Enabling

Participants identified several aspects of their organization’s culture that currently supported their experiences working remotely. Thirty-two participants (leaders: *n* = 18, 51%, IC: *n* = 14, 58%) believed that their organization had a culture that values and supports employees which made them feel supported during their transition to remote work. There was a general perception that people believed good work and productivity came from remote work (leaders: *n* = 14, 40%, IC: *n* = 10, 29%) and working virtually allowed for expertise to be leveraged from all areas of the organization (leaders: *n* = 4, 11%, IC: *n* = 2, 8%). The primary challenge, although only reported by one leader and one individual contributor, of the current culture was the potential envy of remote workers from other employees that could not work remotely and potential perceptions of unfairness, demonstrating that organizational culture can both enable and challenge successful remote work experiences. In addition to the organizational culture, participants also commented that working remotely enabled them to adopt a healthier lifestyle which in turn, aided them in being more successful in their professional and personal domains and perhaps even remaining in the workforce longer (leaders: *n* = 8, 23%, IC: *n* = 10, 42%): “*I think as long as I’m working from home, I think I probably will be more active. And I’m only a couple of years away from retirement [but] if I work from home, I may work a little bit longer*” (JC2803, leader).

#### Challenging

In addition to the positive experiences with remote work, numerous challenges with a remote work environment were reported. Common remote work challenges such as technology and equipment (leaders: *n* = 20, 57%, IC: *n* = 9, 26%) and connectivity issues (leaders: *n* = 17, 49%, IC: *n* = 10, 29%) were reported; however, the challenges went beyond organizational resources. For example, 80% of leaders (*n* = 28) commented on the significant challenge of building and maintaining relationships with employees in a remote environment.

*“Attending to community has been the biggest challenge for celebrations, and for the [bereavement] greetings and making sure people feel part of something beyond just the work that they do. Because I think that’s why we’re so successful at retaining our staff here is we’ve traditionally had really strong communities of people who have been invested in the same industry and they know that they’re all in it for the long run and it’s a really nice thing to have*…*it’s been hard to support my team and for us to support each other”* (AS1611, leader).

Leaders were also concerned with on-boarding new employees remotely and the challenges of succession planning (*n* = 21; 60%) when opportunities for networking and mentoring were perceived to be lower.

*“[It’s been challenging] to, you know, pass on your knowledge and experience and figuring out who’s going to be the best person for doing those sorts of things. When you’re in an office and you’re working together and you’re physically seeing what everybody is capable of and how well they work, it’s easier to me anyway to figure out who’s the best person that can do [the task] or fits in with that particular piece of the role*…*so that’s been tough. And I know for our team, it’s not just been difficult for me from a manager perspective but it’s also being difficult for the individual that’s considering retiring.”* (LA1601, leader)

Although fewer remote work challenges were reported by employees overall, ICs faced many of the same challenges as leaders. Relationships were challenging to build and maintain (*n* = 13; 38%) and work-home interference challenges were posed by the need for a dedicated workspace (*n* = 8; 33%) and the struggle to maintain a healthy work-life balance (*n* = 8; 33%). Indeed, some participants shared their concerns that their attempts to find balance while working at home would be perceived differently than if they were in the office: “[at the office] *if you’re out doing things or whatever, taking a break or running downstairs to grab something to eat, it’s not a big deal. But when you’re at home, it feels like you’re tied to the computer because somebody’s going to see you not there*” (JV2309, IC). The primary challenge that ICs reported more than leaders was the difficulty of staying focused, although it was only reported by a minority of participants (*n* = 6; 25%).

### Remote work moving forward

#### Skills to develop

Continued skill development was identified as an important facilitator of successful remote work. For leaders, increased knowledge in managing virtually (*n* = 19; 54%) and improved technology literacy at all levels of the organization (*n* = 18; 51%) were the primary areas of skill development. When discussing how introducing a shared document system for their team positively impacted their work, a leader highlighted how critical technical skills were to managers when working remotely: “*If you want to continue with virtual work, you would really need to invest in those skills because they’re critical to success*” (TH1310, leader). In reference to the importance of developing skills for maintaining rapport virtually (*n* = 15; 43%), another leader commented: “*I think it’s particularly important when you’re remote because again you need people to feel comfortable reaching out to you and connecting with you right? And they need to be I guess expecting that you’re going to be doing the same and reaching out to them*” (DW1410, leader).

Individual contributor(s) also wanted to see improved management skills in a virtual environment (*n* = 8; 33%) in addition to more personal management skills such as time management (*n* = 14; 58%), communication (*n* = 8; 33%), and self-management (*n* = 6; 25%). One participant quote highlighted the intersection of time management and how that can be impacted by ineffective communication and management from others:

*“Definitely time management skills. Just so that you’re present at work, or so that everyone’s present at work and reachable when they’re supposed to be. And then on the other hand, not working, more than, than you need to be. Like sometimes my manager will put in a request on a Thursday late day. So, then I’ll just be working Thursday afternoon to get it done because I won’t have time to do it on Friday.”* (AW1509, IC)

#### Organizational culture development

As highlighted above, organizational culture is an important foundation for successful remote work and can foster success but also pose significant challenges. Despite participants perceiving their organizational culture to be shifting to be more supportive of remote work practices, approximately half of the participants (*n* = 29; 49%) were concerned that the culture shifts they had witnessed would be reversed when work-from-home orders were lifted. Comments such as these show that organizational changes need to be substantial and longstanding for successful remote work to continue moving forward. Further, although positive changes had occurred, there was still a perceived need to increase trust in remote workers as an organization (leaders: *n* = 19, 54%, IC: *n* = 11, 46%). Participants also wanted a strong “camera-on” culture (leaders: *n* = 10, 29%, IC: *n* = 3, 13%) where people engaged more actively in virtual meetings, turned their webcams on, and continued to foster strong relationships even when working apart. As one participant stated: “*when it’s a smaller subgroup, then let’s use a webcam so you can actually see people’s faces and encourage the cultivation of compassion for one another. Because it’s so easy to get angry with somebody who you only see as a name on the screen right?*” (AS1611, leader). Using tools that approximate face-to-face interactions, participants believed that they could work to overcome many of the connection and conflict challenges posed by working remotely. Lastly, increased remote and hybrid remote work demands an increased respect for employees’ work-life balance and availability (leaders: *n* = 3, 9%, IC: *n* = 1, 4%).

#### Enabling future remote work

Finally, leaders and ICs identified several organizational actions they believed would facilitate a healthy remote work environment when the limitations posed by the COVID-19 pandemic ease. Both leaders and ICs emphasized the need for access to the necessary technology and equipment (leaders: *n* = 26, 74%, IC: *n* = 15, 63%) and improved IT support (leaders: *n* = 9, 26%, IC: *n* = 6, 25%). Clearly and transparently communicating remote work expectations were also important especially when it came to policies and procedures, role expectations, and cost expectations (e.g., setting up a home office).

Although structural supports are necessary, they are not sufficient for a thriving remote workplace. Specifically, both leaders and ICs want to feel heard and that their wants and needs have been considered (leaders: *n* = 21, 60%, IC: *n* = 18, 75%), especially for employees that may adopt a hybrid remote work model and have unique needs compared to fully remote workers (e.g., transporting work equipment). Skills training can be a way to help support the development of the specific skills employees need to succeed in a remote environment (e.g., managing virtually, communicating virtually) (leaders: *n* = 17, 49%, IC: *n* = 7, 29%) and relationship building opportunities can facilitate important connections that can support employees during times of professional isolation (leaders: *n* = 7, 20%, IC: *n* = 7, 29%). Networking also provides the opportunity for contact with other workgroups which can facilitate necessary, and often taken for granted, information sharing: “*we’re really missing that opportunity for the synergies that you can achieve when you’re talking to people. And I think that that’s something that’s really important going forward*” (RF2109, IC).

## Discussion

In March 2020, the COVID-19 pandemic sent millions of workers home, who had to rapidly adapt to the new context and demands of remote work, presenting a unique opportunity to interview these individuals, and to identify and learn from the strategies, behaviors, traits, and skills that helped workers not previously exposed to remote work to be successful. Indeed, the transition to technology-mediated communication typically creates difficulties for individual contributors, leaders, and teams, particularly in the short term (cf. Swaab et al., 2012). To learn more about how employees adapted and provide insights into future practices for remote work, we interviewed a large sample of remote workers, consisting of Leaders and ICs, which was diverse and representative of the Canadian working population in terms of organizational tenure, gender, age, and urban and rural geographic residency.

Research in the past on remote work has examined areas such as who engages in remote work, how interactions differ between remote and non-remote work environments, why individuals and organizations choose to engage in remote work, different kinds of remote work, and the outcomes of remote work (e.g., Bailey and Kurland, 2002; Gajendran and Harrison, 2007; Allen et al., 2015; Golden and Gajendran, 2019). Our study addresses a gap in the literature by considering how employees adjusted to remote work, managed the new communication challenges, and by examining the skills and behaviors they learned to employ to help with this adjustment. Importantly, these employees previously did not have the option to work remotely, or alternatively did not choose to.

From these interviews, we have identified several themes which we will outline further in our discussion; the key skills and behaviors that allowed workers to better succeed in a remote work environment, the enabling aspects like support from management and leadership or the aspects of organization culture that participants perceived supporting their success with remote work and, conversely, the challenges and barriers to success with remote work (refer Table 3). We examined how these experiences can inform remote work moving forwards such as through identifying how leadership can better support remote workers, aspects of organizational culture that participants hoped to see changed or improved within their organization, and future skillsets and training opportunities which would be beneficial to other organizations adopting remote work in the future. Indeed, some research suggests approximately 40% of jobs in Western countries such as Canada could be done by remote workers (Deng et al., 2020; Gallacher and Hossain, 2020), or up to 56% in the United States (Global Workplace Analytics, 2021). In wake of the substantial changes the COVID-19 pandemic brought, our findings present a more up to date understanding of remote work that better captures the new and unique perspectives from many workers whom, though previously unexperienced with remote work, are now well acquainted with it. Finally, we provide a commentary on the experiences that participants have had and the lessons they’ve learned to provide guidance on future research and training, and identify what organizational cultural shifts may be necessary to ensure remote work can be successful in the future.

**TABLE 3:** Table of recommendations.

Challenge/problem	Consequence	Our recommendations
Unclear remote work policies	– Feelings of uncertainty and unfairness – Performance management challenges – Scheduling challenges	– Create clear and unambiguous policies around remote work eligibility – Policies should: ° Consider employees wants and needs ° Only include restrictions strictly necessary to achieve goals ° Outline remote work frequency and performance expectations (e.g., timelines, success metrics, benchmarks)
Concerns of negative treatment of remote workers; leadership not understanding the work remote workers do, remote workers worried about not being promoted fairly, etc.	– Remote workers feeling unsupported by their organization – Undervaluing remote workers, not having accurate perceptions and recognition of their achievements – Undermines commitment, creates rifts within and between teams, and strains leader-member relationships – Also undermines remote work itself, potentially resulting in a full-scale return to office	– Lines of communication between remote and non-remote workers (especially leadership and management) must be actively facilitated ° Routinely check, address, and clarify assumptions of what remote workers have accomplished – Organizational leadership must also dismantle negative stereotypes or rumors of unfair/preferential treatment ° Emphasize how all contribute toward success; share success stories from both remote and non-remote workers
Management and leadership being ‘out of touch,’ not accurately understanding remote worker needs	– Poor performance, satisfaction, and well-being in a remote work environment due to poor leadership and a lack of role modeling	– Remote workers need to trust that leadership and policy makers can understand the challenges remote workers face. ° Train leaders in remote work tools (e.g., Use of teleconferencing tools), as well as specific training in how to manage remote workers (e.g., how to hold a successful virtual team meeting)
Positive work relationships and interactions are difficult to maintain in a remote work environment, and networking and building relationships can be a major challenge for newer employees	– Higher risks of remote workers feeling isolated or lonely – Perceptions of being socially isolated can lead to increased burn-out and attrition – Lack of learning, development, promotions, and advancement in the organization leading to a lack of growth and greater attrition	– Provide socializing opportunities analogous to what non-remote workers experience. ° Dedicated informal meeting and socializing time (replicating water-cooler/coffee shop talk) ° Networking opportunities with other individuals at the organization. – Over-engaging in these activities, or virtual meetings in general, can lead to burn-out or disengagement – Avoid making ‘informal’ events mandatory to attend, or holding them extremely frequently – Avoid blanket ‘Camera On’ policies; only use them if they serve a genuine purpose – Use leaner forms of communication (e.g., email) if they would suffice in lieu of videoconferencing

### Key traits, skills, and behaviors

Most of our participants were able to identify skills, habits, or behaviors that over the course of their remote work experience had helped them to be more successful. Some of these skills and behaviors participants reported they had before the switch to remote work and were found to be of benefit to them, such as strong technological communication proficiency making the transitional period much smoother overall. Other skills and behaviors were learned or adopted after in order to meet new contextual demands, such as workers needing to find various ways to not depend on paper hard copies of documents, or how to lead or manage teams with no ability to meet face to face. These successes reflect over 12 months (at the time of data collection) of trial and error as participants figured out how to best achieve success in an environment where, due to work from home mandates issued from the government, they had no other alternative but to try and make it work. It should not come as a surprise that if there are alternative work environments or arrangements available, future workers may be less motivated to try and persevere and overcome such an initial adjustment period. Thus, it is extremely important that we identify the skills, traits, and behaviors that lead to success for remote workers so that they can be used to inform necessary training, guidelines, and policy to support remote workers in the future.

Given the level of both perceived and actual success around remote work, it is clear that remote working presents a favorable environment for achieving success for many (but not all) individuals, with 66% of participants in our sample reporting management should listen to their input around continuing with remote work. The general preference for remote work continuing likely stems from the various benefits workers reported that working from home provided them; for example, 58% of workers reported remote work allowed them to do get more work done by increasing the amount of “deep focus” time they had without interruption from co-workers or by removing the time spent commuting to and from work each day, and 31% of participants reported that their lifestyle had become healthier in various ways as a result of the shift to remote work. However, many participants also reported that working from home introduced a greater availability of non-work distractions which required specific distraction management skills to overcome.

This seemingly self-contradicting result between themes could be explained by Trait Activation Theory, which as discussed earlier, suggests that observable outcomes are a product of the environment and context influencing which traits are activated, and how they manifest (Tett and Guterman, 2000). For example, even though the remote work environment presents multiple other cues for behaviors to manifest (e.g., being distracted by entertainment such as TV or Netflix, or by family members), some participants expressed that they were able to achieve higher productivity specifically because of the remote work creating an environment where they could work without interruption from co-workers or the other distractions present in their office environments. Grelle and Popp (2021) outlined several traits and characteristics that may be of particular importance to remote workers given the differences in a traditional work environment compared to remote work. For example, they note how communication occurring strictly through technology significantly alters the social environment and relationship building opportunities available, which may result in more extroverted individuals feeling isolated; and how too little work focus may result in one being distracted, but too much may contribute to overwork and burnout. Thus, different environments may require different skills or behaviors by remote workers in order to succeed (e.g., needing to be more cognizant of possible distractions if you do not have a dedicated workspace for remote working).

Interestingly, within our sample many participants indicated that they found improved success in their remote work by simply replicating the schedule and habits they had when they were still commuting into work in person, and by adhering to their normal work schedule. This was usually some combination of what one would normally do to ‘get ready for the day,’ such as showering, having breakfast, or putting on work appropriate attire before sitting down, in order to get them into the same mindset that they would be in if they were going to work ‘in person.’ The majority of participants (71%) mentioned if they would have left the office at a specific time, they made a conscious effort to disconnect from work at the same time, sometimes going as far as physically unplugging work issued devices in order to do so. Thus, while remote workers are influenced by the environment they are in, they can also change the environment itself or engage in behaviors aimed at creating a more successful remote work experience for themselves.

Several findings were consistent with leader-member exchange theory (LMX; Erdogan and Bauer, 2014), which emphasizes the unique relationships leaders have with each of their direct reports. The ICs we interviewed substantially felt that leader behaviors such as being flexible and providing ample support were important aspects contributing to their success with remote work. Half of ICs reported that their leaders had been engaging in what we labeled ‘check-in’ behaviors, where leaders would much more frequently communicate with direct reports and see how individual members of their teams were doing. Half of leaders reported that they taking additional efforts to be deliberate and intentional about their communications with others, ensuring that they were being mindful of which virtual tools they used to communicate what messages to not overwhelm or waste unnecessary time in meetings.

Finally, finding ways to reduce perceived team virtuality (Handke et al., 2021) – and feeling closer and more connected even when communicating through technology – was important. Restrictions around face-to-face gatherings also led to increased communication creativity, and teams finding ways to stay in touch that they had not ever considered before. Many took efforts to maintain non-work interactions with their co-workers; some teams even reported this became part of a structured routine, where they had formally designated timeframes solely dedicated toward socialization or other casual interactions with each other. Growing familiarity and comfort with remote work technology, especially virtual conferencing tools like Zoom, helped with workers using these tools for these more informal engagements, and we encourage remote workers – both Leaders and ICs – to continue taking deliberate effort to ensure they communicate openly and regularly, and remain connected with their colleagues.

### Remote work enablers and challenges

Working remotely had not previously been the norm for the majority of our participants, but many found themselves surprised at how attitudes (both theirs and of their coworkers) toward remote work changed to be more positive. Many cited this shift as a function of the effective behaviors or skills they developed as they gained more experience with working remotely. Some participants perceived that their success in remote work was partially a result of not being negatively impacted or hindered by the overall change to the situation. Indeed, some individuals noted that after some discomfort around the initial adjustment, they realized their day-to-day tasks were functionally identical as to when they were working in an office, and required little to no adjustment or learning of new skillsets and capabilities. Overall, the majority of participants felt positively toward remote work, and desired their organization to listen to their needs and wants of continuing some degree of remote work in the future, findings which are not unique to our study alone (Aczel et al., 2021; Babapour Chafi et al., 2022).

In addition to the work-related benefits of remote work, participants expressed numerous non-work-related benefits. Many participants indicated that as a function of the reduced commute time, they were able to tackle errands at home (e.g., doing chores and household errands), spend more quality time with loved ones (especially those that were also now remote working), or spend more time attending to their physical health (e.g., going for walks) which further supported their productivity during work hours. Some participants reported commuting alone previously took up multiple hours of their time, which could now be used to attend personal needs (leisure or health) instead. By being able to optimize time spent during their day, many participants perceived they had an overall healthier lifestyle than before working remotely which made the remote work experience more amenable to them. Indeed, other research has shown that when properly implemented, remote work can improve feelings of autonomy and work-life balance (Babapour Chafi et al., 2022). At an organizational culture level, mentions of how appreciated workers were, how the work they were doing in face of the pandemic environment was important, and the organization’s mission of providing healthcare to Canadians was valued, were all mentioned as being important factors to participants’ success in the shift to remote work.

However, despite the general positivity toward remote work, there were many challenges to overcome, and many of these were identified to be on-going issues or concerns. Many participants reported that the lack of a dedicated workspace as a major issue, often not having the option to have one due to limited space at home or the costs of creating a home office being too great. Many participants, particularly leaders, had concerns about work-life balance and managing their work schedules, some with comments around finding themselves working when they should not have been (e.g., checking for a response to an email they sent before going to bed). It was not uncommon to hear participants express they were struggling with work spilling over into their personal lives, or with their personal lives interrupting their work (e.g., pets, spouses, or childcare-related issues disrupting virtual meetings), which is similar to other findings in research (Toscano and Zappalà, 2021).

Additionally, communication, both in terms of socialization as well as in terms of work duties and keeping up to date on projects with other remote workers or across teams, was another notable challenge. Although a minority, some ICs also felt they received poor communication and direction from organizational leaders and perceived a lack of management visibility. Almost half of Leaders in our sample reported that performance management or the perceptions and uncertainty surrounding performance management was a major challenge for them. In contrast, some participants expressed that while they want to continue with remote work in the future, they would not desire to do so if their organization implemented monitoring software on their work devices or had supervisors that micro-managed them. In line with this, around half of the participants (both Leaders and ICs) identified that they had major concerns around unfair treatment or stigma toward remote workers. Some individuals were worried about being less likely to be promoted than a non-remote worker in the future, and others expressed concerns that individuals choosing to remote work in the future would be perceived as slacking off continually and being lazy. Leaders expressed they had faced significant challenges around being able to maintain relationships, build new ones, and with the on-boarding of new employees or conversely, and with succession planning to account for voluntary and involuntary turnover. Finally, many remote workers reported difficulties around fulfilling socialization-related needs, such as maintaining relationships and networking with others were common in our participants, echoing other research on the social effects of the pandemic and remote work (Van Zoonen and Sivunen, 2021; Van Zoonen et al., 2021).

Many of the challenges outlined by our participants point to key areas that need to be addressed and supported for successful remote work moving forward. In particular, we highlight the need for clear and consistent communication around remote work expectations and policy, as well as the skills necessary to facilitate successful remote work.

### Informing remote work moving forward

The majority of participants reported that they desired to continue with remote work in the future. However, at the time of our interviews, the organization had not yet provided any guidance on whether remote work would continue to any degree in the future. In addition to previously mentioned concerns around unfair treatment of remote workers, some participants stated that some decision makers at the organization were resistant to the idea of adopting remote work in the future, even when faced with internal data showing the overall successes and benefits of remote work. Approximately half of leaders reported that they needed to be able to more clearly communicate remote work expectations to their direct reports, which they would be unable to do without formal guidance from senior leadership. However, when participants spoke about their desires for remote work in the future, there was a diverse range in preferences; some participants desired the discretion to decide to work remotely and to work in-person, and for others it was the ability to simply work remotely permanently. Rectifying these two competing demands of needing to provide clear, fair expectations and guidance to workers and leaders, and of employee trust and empowerment, will be key for sustainable success in remote work. We interpret the challenges as stemming from a low clarity between personal life and work life, whether the choice to work remotely or not is truly a choice, and from both ICs and Leaders not having a clear understanding of what is being expected of remote workers. Below, we outline several recommendations for rectifying these areas, based in the data we have gathered from the experiences of our participants.

#### Recommendations

First, remote workers should have the same confidence as non-remote workers in knowing when they begin and finish their work for the day, of what they are expected to accomplish, and be trusted to be given the freedom to choose where and how they complete their work. Robert Half, a Canadian recruitment agency, found that over half of survey respondents reported they would seek out a new employer if their current employer required them to return to their offices for a full 5 days per week (Robert Half, 2022). Going forward, organizations will need to ensure they have clear plans to communicate their remote work policies and expectations to both prospective and current workers. However, research on Boundary Theory indicates that a combination of support from an organization to choose to engage in remote work, plus an individual’s *ability to control* the separations between their work and family life leads to more positive outcomes than those that have little ability to do so (Kossek et al., 2006, 2012). Thus, we emphasize that the positive benefits here are not necessarily from the separation between work and home itself (refer Kreiner, 2006) but from the freedom to make one’s working conditions more optimal to one’s individual needs and preferences. Echoing these findings, despite broadly positive attitudes toward remote work in our sample, around half of participants reported that they would still desire the organization to retain physical office space for a wide variety of specific needs (e.g., socialization, access to resources like printers, separation from work and home, etc.). Organizations will need to balance individual discretion with challenges around coordination. Leadership and policy makers should consider what at minimum is necessary for their organizations to coordinate and allow for broad individual discretion outside of those necessities. For example, an organization could consider allowing all employees to choose whether to work in office or work remotely on specific days of the week (e.g., able to work remotely or in person on Wednesdays, and Thursdays), and require approval to work remotely outside of those days. However, it should be kept in mind that strict policies will undermine employees’ sense of autonomy, and increased autonomy was a major desire reported by many of our respondents.

Second, leadership must ensure they are working systematically to remove organizational culture barriers or stigmas toward remote work and remote workers, such as negative stereotypes, and address any fears of unfair treatment. Having tangible successes, such as showing figures of how remote workers are contributing toward metrics or common goals at the organization, or showcasing exemplar achievements and outcomes that remote workers or remote working teams achieved, may help address organizational culture and performance management-related concerns. These could be sent out in organizational communications such as newsletters to both remote and non-remote teams. Leaders could also ask individuals or teams that have performed exemplary work whether they would like acknowledgment via emails or other forms of announcements. As well, data on performance should be collected so that objective figures of remote team effectiveness can be presented and made readily available to management and other decision makers in the organization.

Third some of our participants expressed concerns that future decisions around remote work would not be well informed or effective. This was due to perceptions that individuals occupying decision making positions at the organization often lacked basic proficiency in using remote work technology. Of note is that almost twice as many Leaders as ICs identified technological literacy as being a helpful skill for communication during remote work. Additionally, perhaps self-aware of the issue of technological proficiency, more Leaders than ICs felt technological skills needed to be improved across the entire organization going forward. Remote workers need to feel that that leaders and policy makers understand the demands and contexts that remote workers face, and can effectively communicate with and manage them, which is significantly more difficult if leaders are unable to operate technology proficiently. Leaders must have the skillsets and the confidence that they are prepared to manage remote workers, receiving training if necessary to do so. Some of the skillsets both ICs and leaders identified as needing improvement for remote work to succeed in the future were time management skills, communication skills, virtual ‘soft skills,’ self-management skills, and training on how to maintain rapport virtually.

Fourth, building, emphasizing, and maintaining positive work relationships between managers and their direct reports is an important determinant of organizational commitment, job satisfaction, and job performance, and the quality of these relationships may be particularly important for remote workers (Golden and Veiga, 2008). When making efforts to maintain social interactions, theories such as Media Richness theory (Daft and Lengel, 1986) in conjunction with our findings can inform further recommendations for organizations. Tools like Zoom that are high in media richness may help with feelings of closeness and social presence of co-workers (Rice, 1992). Richer forms of media which increase perceived social presence can help create higher quality relationships and enhance work commitment (Workman et al., 2003). Indeed, participants in our sample indicated that informal team meetings and activities were helpful at maintaining morale, well-being, and overall team functioning. Considerations should be made as to whether organizational sponsored events (e.g., town halls, conferences, or announcements) are being adequately communicated to, and made accessible to, remote workers. If possible, in-person events should be live streamed or recorded so that remote workers are able to engage and opportunities to facilitate two-way communication should be encouraged. For example, additional efforts should be made so that remote workers who wish to participate asynchronously are still able to participate in any Q&A sessions to the same extent that individuals attending events in-person are, for example by allowing remote workers to send their questions in advance to moderators or panelists.

However, overuse of virtual meetings, and especially ‘Camera On’ policies may become emotionally draining and fatiguing over time (Bailenson, 2021; Shockley et al., 2021). To minimize deleterious effects, we recommend that leaders consider whether having a camera on in any given meeting serves a genuine purpose, and whether a leaner form of communication (e.g., email) would be sufficient for the message they are trying to communicate.

## Conclusion

Following the COVID-19 pandemic there was a rapid expansion in the number of individuals engaging in remote work. Many of these remote workers were entirely new to a remote working arrangement, and included individuals who would not otherwise be drawn to remote work or potentially outright opposed to the idea of engaging in remote work. By interviewing a broad sample of Individual Contributors and Leaders at an organization, we sought to capture the experiences of these individuals as they grew accustomed to remote work. We looked at skills, characteristics, behaviors, and situations that contributed to their successes, as well as aspects of remote work that posed challenges for them. Across both Individual Contributor and Leader samples, we synthesized common themes into recommendations backed by previous theory relevant to remote work. Clear communication will be important factors, and leaders should craft policy that empowers individuals to feel like they have genuine control over their choice to engage in remote work. Shared understanding of what is expected of remote workers must be held by both remote workers and their leaders, and remote workers must be carefully supported by leadership and management to ensure an organization’s culture eliminates unfounded biases held against remote workers, and that fears and concerns around unfair treatment are thoroughly addressed.

Our study contributes to the rapidly expanding body of work studying the changes and impacts of remote work experienced during the COVID-19 pandemic. We adhered to qualitative methodology that has been employed in the past, with rigorous efforts employed to ensure the themes explored and analyzed genuinely reflected the experiences of the participants. However, while our sample was overall diverse in terms of tenure, gender, age, and urban and rural geographic residency, participants were sourced from one single healthcare organization, operating in one country, which reduces the generalizability of our sample across organizational and national cultural boundaries. Additionally, in other for-profit organizations where executives and leadership have a fiscal responsibility to generate profits and returns for shareholders, different goals could affect motivations, incentives, behaviors, and overall organization culture. However, our recommendations address the foundations and fundamentals for individual, team, and organizational culture focused outcomes. Thus, even if the overall goals of an organization may vary, our recommendations are built upon research and theory which have been shown to be important in a wide variety of contexts.

Finally, we fully recognize many areas which are challenges in one organization or team could be sources of strength and success in another. Within our own sample we saw a diverse range of opinions around whether aspects of remote work were considered successful or not, and what some report as challenges others saw as unanticipated boons. For example, while some participants reported that maintaining rapport with their team members was a significant challenge, other participants reported that their team became more comfortable with being emotionally vulnerable with each other, leaned on each other for support, and trusted each other more than before the switch to remote work. There still remains a wealth of opportunity for researchers to examine factors that explain further nuances in remote work, such as across aspects of organizational culture (Cameron, 1985; Chapman et al., 2018), team interactions (Handke et al., 2021), and individual differences like personality types (Lee et al., 2005), and for researchers to translate new and recent findings into recommendations for practitioners.

## Data availability statement

The datasets presented in this article are not readily available because individual and aggregated data cannot be released in order to protect participant confidentiality. Enquiries regarding the datasets should be directed to jbhenke@ucalgary.ca.

## Ethics statement

The studies involving human participants were reviewed and approved by the Conjoint Research Ethics Board (REB21-1830) at the University of Calgary, and the Health Systems Access board at the participating organization. The participants provided their verbal informed consent to participate in this study as approved by the ethics boards. Written informed consent for participation was not required for this study in accordance with the national legislation and the institutional requirements.

## Author contributions

TO’N and SJ contributed to the conceptual components of the study and the study and interview guide design. SJ lead the data collection process and wrote the method and results. SJ and JH interviewed the participants and analyzed the interview transcripts. JH wrote the introduction, discussion, and conclusion with input from SJ and TO’N. All authors read and reviewed the final version of the manuscript, and approved its submission.

## References

[B1] Aczel B. Kovacs M. Van Der Lippe T. Szaszi B. (2021). Researchers working from home: Benefits and challenges. *PLoS One* 16:e0249127. 10.1371/journal.pone.0249127. 33765047 PMC7993618

[B2] Allen T. D. Golden T. D. Shockley K. M. (2015). How effective is telecommuting? Assessing the status of our scientific findings. *Psychol. Sci. Public Interest* 16 40–68. 10.1177/1529100615593273 26403188

[B3] Anderson D. Kelliher C. (2020). Enforced remote working and the work-life interface during lockdown. *Gender Manag.* 35 677–683. 10.1108/GM-07-2020-0224

[B4] Babapour Chafi M. Hultberg A. Bozic Yams N. (2022). Post-pandemic office work: Perceived challenges and opportunities for a sustainable work environment. *Sustainability* 14:294. 10.3390/su14010294

[B5] Bailenson J. N. (2021). Nonverbal overload: A theoretical argument for the causes of Zoom fatigue. *Technol. Mind Behav.* 2. 10.1037/tmb0000030

[B6] Bailey D. E. Kurland N. B. (2002). A review of telework research: Findings, new directions, and lessons for the study of modern work. *J. Organ. Behav.* 23 383–400. 10.1002/job.144

[B7] Baudot L. Kelly K. (2020). *A Survey of Perceptions of Remote Work and Work Productivity in the United States during the COVID-19 Shutdown.* 10.2139/ssrn.3646406 Rochester, NY: SSRN.

[B8] Braun V. Clarke V. (2012). “Thematic analysis,” in *APA Handbook of Research Methods in Psychology: Research Designs: Quantitative, Qualitative, Neuropsychological, and Biological.* eds Cooper H. Camic P. M. Long D. L. Panter A. T. Rindskopf D. Sher K. J. Vol. 2. (Washington, DC: American Psychological Association), 57–71.

[B9] Cameron K. S. (1985). “Cultural congruence, strength, and type: relationships to effectiveness,” in *Paper Presented st the ASHE 1985 Annual Meeting Paper*, (Chicago, IL).

[B10] Chapman D. S. Reeves P. Chapin M. (2018). A lexical approach to identifying dimensions of organizational culture. *Front. Psychol.* 9:876. 10.3389/fpsyg.2018.00876 29922200 PMC5996186

[B11] Charlier S. D. Stewart G. L. Greco L. M. Reeves C. J. (2016). Emergent leadership in virtual teams: A multilevel investigation of individual communication and team dispersion antecedents. *Leadersh. Q.* 27 745–764. 10.1016/j.leaqua.2016.05.002

[B12] Cheshin A. Rafaeli A. Bos N. (2011). Anger and happiness in virtual teams: Emotional influences of text and behavior on others’ affect in the absence of non-verbal cues. *Organ. Behav. Hum. Decis. Process.* 116 2–16. 10.1016/j.obhdp.2011.06.002

[B13] Clarke V. Braun V. (2013). Teaching thematic analysis: Overcoming challenges and developing strategies for effective learning. *Psychologist* 26 120–123.

[B14] Daft R. L. Lengel R. H. (1986). Organizational information requirements, media richness and structural design. *Manag. Sci.* 32 554–571. 10.1287/mnsc.32.5.554 19642375

[B15] Deng Z. Morissette R. Messacar D. (2020). Running the Economy Remotely: Potential for Working from Home during and after COVID-19. StatCan COVID-19 : Data to Insights for a Better Canada no 00026. Statistics Canada Catalogue No. 45280001. Ottawa: Statistics Canada.

[B16] Dennis A. R. Fuller R. M. Valacich J. S. (2008). Media, tasks, and communication processes: A theory of media synchronicity. *MIS Q.* 32 575–600. 10.2307/25148857

[B17] Erdogan B. Bauer T. N. (2014). “Leader-member exchange (LMX) theory: The relational approach to leadership,” in *The Oxford Handbook of Leadership and Organizations*, ed. Day D. V. (Oxford: Oxford University Press), 407–433. 10.1093/oxfordhb/9780199755615.013.020

[B18] Eurofound (2021). *European Industrial Relations Dictionary.* Available online at: https://www.eurofound.europa.eu/observatories/eurwork/industrial-relations-dictionary/telework (accessed April 22, 2022).

[B19] Gajendran R. S. Harrison D. A. (2007). The good, the bad, and the unknown about telecommuting: Meta-analysis of psychological mediators and individual consequences. *J. Appl. Psychol.* 92:1524. 10.1037/0021-9010.92.6.1524 18020794

[B20] Gallacher G. Hossain I. (2020). Remote work and employment dynamics under COVID-19: Evidence from Canada. *Can. Public Policy* 46 S44–S54. 10.3138/cpp.2020-026 PMC7971424 38629973

[B21] Gartner (2020). *12% of Organizations Are Highly Prepared for COVID.* Stamford, CT: Gartner.

[B22] Gilson L. Costa P. O’Neill T. A. Maynard M. T. (2021). Putting the “TEAM” back into virtual teams. *Organ. Dyn.* 50:100847. 10.1016/j.orgdyn.2021.100847

[B23] Glikson E. Erez M. (2013). Emotion display norms in virtual teams. *J. Pers. Psychol.* 12 22–32. 10.1027/1866-5888/a000078

[B24] Global Workplace Analytics (2021). *Latest Work-at-Home/Telecommuting/Remote Work Statistics.* Available online at: https://globalworkplaceanalytics.com/telecommuting-statistics (accessed February 10, 2022).

[B25] Golden T. D. Gajendran R. S. (2019). Unpacking the role of a telecommuter’s job in their performance: Examining job complexity, problem solving, interdependence, and social support. *J. Bus. Psychol.* 34 55–69. 10.1007/s10869-018-9530-4

[B26] Golden T. D. Veiga J. F. (2008). The impact of superior–subordinate relationships on the commitment, job satisfaction, and performance of virtual workers. *Leadersh. Q.* 19 77–88. 10.1016/j.leaqua.2007.12.009

[B27] Grelle D. Popp E. (2021). Considering the interaction of individual differences and remote work contexts. *Ind. Organ. Psychol.* 14 244–247. 10.1017/iop.2021.51

[B28] Handke L. Costa P. L. Klonek F. E. O’Neill T. A. Parker S. K. (2021). Team perceived virtuality: An emergent state perspective. *Eur. J. Work Organ. Psychol.* 30 624–638. 10.1080/1359432X.2020.1806921

[B29] Kossek E. E. Lautsch B. A. Eaton S. C. (2006). Telecommuting, control, and boundary management: Correlates of policy use and practice, job control, and work–family effectiveness. *J. Vocat. Behav.* 68 347–367. 10.1016/j.jvb.2005.07.002

[B30] Kossek E. E. Ruderman M. N. Braddy P. W. Hannum K. M. (2012). Work–nonwork boundary management profiles: A person-centered approach. *J. Vocat. Behav.* 81 112–128. 10.1016/j.jvb.2012.04.003

[B31] Kreiner G. E. (2006). Consequences of work-home segmentation or integration: A person-environment fit perspective. *J. Organ. Behav.* 27 485–507. 10.1002/job.386

[B32] Kristof A. L. (1996). Person-organization fit: An integrative review of its conceptualizations, measurement, and implications. *Pers. Psychol.* 49 1–49. 10.1111/j.1744-6570.1996.tb01790.x

[B33] Lee K. Ashton M. C. de Vries R. E. (2005). Predicting workplace delinquency and integrity with the HEXACO and five-factor models of personality structure. *Hum. Perform.* 18 179–197. 10.1207/s15327043hup1802_4

[B34] Lund S. Madgavkar A. Manyika J. Smit S. (2020). *What’s Next for Remote Work: An Analysis of 2,000 Tasks, 800 Jobs, and Nine Countries.* San Francisco, CA: McKinsey Global Institute, 1–13.

[B35] Maduka N. S. Edwards H. Greenwood D. Osborne A. Babatunde S. O. (2018). Analysis of competencies for effective virtual team leadership in building successful organisations. *Benchmarking Int. J.* 25 696–712. 10.1108/BIJ-08-2016-0124

[B36] McLarnon M. J. O’Neill T. A. Taras V. Law D. Donia M. B. Steel P. (2019). Global virtual team communication, coordination, and performance across three peer feedback strategies. *Can. J. Behav. Sci.* 51:207. 10.1037/cbs0000135

[B37] Mitchell A. Brewer P. E. (2021). Leading hybrid teams: Strategies for realizing the best of both worlds. *Organ. Dyn.* 100866. 10.1016/j.orgdyn.2021.100866

[B38] O’Neill T. A. Hambley L. A. Bercovich A. (2014). Prediction of cyberslacking when employees are working away from the office. *Comput. Hum. Behav.* 34 291–298. 10.1016/j.chb.2014.02.015

[B39] Raghuram S. Hill N. S. Gibbs J. L. Maruping L. M. (2019). Virtual work: Bridging research clusters. *Acad. Manag. Ann.* 13 308–341. 10.5465/annals.2017.0020

[B40] Rice R. E. (1992). Task analyzability, use of new media, and effectiveness: A multi-site exploration of media richness. *Organ. Sci.* 3 475–500. 10.1287/orsc.3.4.475 19642375

[B41] Robert Half (2022). *More Than Half of Workers in Canada Would Rather Quit Than Return to the Office Full Time, Robert Half Research Shows.* Menlo Park, CA: Robert Half.

[B42] Rofcanin Y. Anand S. (2020). Human relations virtual special issue: Flexible work practices and work-family domain. *Hum. Relat.* 73 1182–1185. 10.1177/0018726720935778

[B43] Roulin N. Bourdage J. S. Hamilton L. K. O’Neill T. A. Shen W. (2021). Emerging research in industrial–organizational psychology in Canada. *Can. J. Behav. Sci.* 53:91. 10.1037/cbs0000274

[B44] Shockley K. M. Gabriel A. S. Robertson D. Rosen C. C. Chawla N. Ganster M. L. (2021). The fatiguing effects of camera use in virtual meetings: A within-person field experiment. *J. Appl. Psychol.* 106:1137. 10.1037/apl0000948 34423999

[B45] Swaab R. I. Galinsky A. D. Medvec V. Diermeier D. A. (2012). The communication orientation model: Explaining the diverse effects of sight, sound, and synchronicity on negotiation and group decision-making outcomes. *Pers. Soc. Psychol. Rev.* 16 25–53. 10.1177/1088868311417186 21846835

[B46] Tett R. P. Guterman H. A. (2000). Situation trait relevance, trait expression, and cross-situational consistency: Testing a principle of trait activation. *J. Res. Pers.* 34 397–423. 10.1006/jrpe.2000.2292

[B47] Toscano F. Zappalà S. (2021). Overall job performance, remote work engagement, living with children, and remote work productivity during the COVID-19 pandemic. *Eur. J. Psychol. Open* 80 133–142. 10.1024/2673-8627/a000015

[B48] Van Zoonen W. Sivunen A. E. (2021). The impact of remote work and mediated communication frequency on isolation and psychological distress. *Eur. J. Work Organ. Psychol.* 1–12. Online ahead of print.

[B49] Van Zoonen W. Sivunen A. Blomqvist K. Olsson T. Ropponen A. Henttonen K. (2021). Factors influencing adjustment to remote work: Employees’ initial responses to the covid-19 pandemic. *Int. J. Environ. Res. Public Health* 18:6966. 10.3390/ijerph18136966 34209796 PMC8297254

[B50] Varty C. T. O’Neill T. A. Hambley L. A. (2017). “Leading anywhere workers: A scientific and practical framework,” in *Anywhere Working and the New Era of Telecommuting*, eds Blount Y. Gloet M. (Hershey, PA: IGI Global), 47–88. 10.4018/978-1-5225-2328-4.ch003

[B51] Workman M. Kahnweiler W. Bommer W. (2003). The effects of cognitive style and media richness on commitment to telework and virtual teams. *J. Vocat. Behav.* 63 199–219. 10.1016/S0001-8791(03)00041-1

[B52] Yin R. K. (2015). *Qualitative Research from Start to Finish.* New York, NY: Guilford publications.

